# The domain architecture of large guanine nucleotide exchange factors for the small GTP-binding protein Arf

**DOI:** 10.1186/1471-2164-6-20

**Published:** 2005-02-17

**Authors:** Barbara Mouratou, Valerie Biou, Alexandra Joubert, Jean Cohen, David J Shields, Niko Geldner, Gerd Jürgens, Paul Melançon, Jacqueline Cherfils

**Affiliations:** 1 Laboratoire d'Enzymologie et Biochimie Structurales, CNRS, avenue de la Terrasse, 91198 Gif sur Yvette cedex, France; 2 Centre de Génétique Moléculaire, CNRS, Gif-sur-Yvette, France; 3 Department of Cell Biology, University of Alberta, Edmonton, Canada; 4 Center of Plant Molecular Biology, Universitaet Tuebingen, Tuebingen, Germany; 5 Plant Biology Laboratory, The Salk Institute for Biological Studies, La Jolla, USA

## Abstract

**Background:**

Small G proteins, which are essential regulators of multiple cellular functions, are activated by guanine nucleotide exchange factors (GEFs) that stimulate the exchange of the tightly bound GDP nucleotide by GTP. The catalytic domain responsible for nucleotide exchange is in general associated with non-catalytic domains that define the spatio-temporal conditions of activation. In the case of small G proteins of the Arf subfamily, which are major regulators of membrane trafficking, GEFs form a heterogeneous family whose only common characteristic is the well-characterized Sec7 catalytic domain. In contrast, the function of non-catalytic domains and how they regulate/cooperate with the catalytic domain is essentially unknown.

**Results:**

Based on Sec7-containing sequences from fully-annotated eukaryotic genomes, including our annotation of these sequences from Paramecium, we have investigated the domain architecture of large ArfGEFs of the BIG and GBF subfamilies, which are involved in Golgi traffic. Multiple sequence alignments combined with the analysis of predicted secondary structures, non-structured regions and splicing patterns, identifies five novel non-catalytic structural domains which are common to both subfamilies, revealing that they share a conserved modular organization. We also report a novel ArfGEF subfamily with a domain organization so far unique to alveolates, which we name TBS (TBC-Sec7).

**Conclusion:**

Our analysis unifies the BIG and GBF subfamilies into a higher order subfamily, which, together with their being the only subfamilies common to all eukaryotes, suggests that they descend from a common ancestor from which species-specific ArfGEFs have subsequently evolved. Our identification of a conserved modular architecture provides a background for future functional investigation of non-catalytic domains.

## Background

Guanine Nucleotide Exchange Factors (GEFs) are obligatory components of signaling cascades regulated by small GTP-binding proteins (called small G proteins hereafter). Their biochemical activity is to stimulate the dissociation of the tightly bound GDP nucleotide from the small G protein in response to cellular signals. Thereby, they favor the binding of the more abundant cellular GTP, organizing the active conformation of the small G protein which can recruit its effectors (reviewed in [1]). Each small G protein family features its own ensemble of GEFs characterized by a conserved catalytic domain responsible for nucleotide exchange, which is generally combined with non-catalytic domains that define the spatio-temporal conditions of activation. In the case of small G proteins of the Arf family, which are major regulators in membrane trafficking (reviewed in [2]), the exchange domain is a conserved module of ~200 amino acids called the Sec7 domain [3]. Its biochemical (reviewed in [4]) and structural [5,6] mechanisms have been investigated in detail. Remarkably, the Sec7 domain is the only domain that is conserved in all ArfGEFs (reviewed in [7,8]) and it is to some extent interchangeable between species [9]. In contrast, little is known about the functions of the other domains, which are likely to determine intracellular localization of ArfGEFs and their responsiveness to specific signals.

As for most small G proteins, Arf family members are outnumbered by ArfGEFs in many species. In humans for instance, 5 Arf proteins have been identified, and there are at least 13 proteins carrying a Sec7 domain, of which most have been characterized as *bona fide *ArfGEFs (reviewed in [7,8]). Thus an individual Arf protein may be activated by more than one GEF, emphasizing that essential aspects in building up the Arf responses may be encoded by the modular architecture of their GEFs. Sequence similarity in the non-catalytic regions forms the basis for the classification of ArfGEFs into subfamilies. 8 subfamilies are currently identified in eukaryotes with sizes ranging from small (~40–80 kD including CYH, EFA6 and FBS), to medium (~100–150 kD, including BRAG/LONER, SYT1, SYT2) and large (~170–200 kD) ArfGEFs (reviewed in [7,8]). Large ArfGEFs comprise two subfamilies which we will refer to as the BIG and GBF subfamilies after the name of their human representatives. The GBF subfamily includes human GBF1 [10], Arabidopsis GNOM [11] and Saccharomyces Gea1 and Gea2 [12], the BIG subfamily human BIG1 and BIG2 [13,14] and yeast Sec7p [15]. An additional subfamily called RalF is found in Rickettsie and Legionella bacteria, likely acting on an host Arf pathway [16]. Analysis of the CYH and EFA6 subfamilies, present only in multicellular animals, and that of the large ArfGEFs, found in all eukaryotes, have yielded most of the functional data currently available. CYH and EFA6 are active on Arf6 at the plasma membrane where they may function in the crosstalk of membrane traffic, cytoskeleton dynamics and signalling in endosomal pathways (reviewed in [17]). Most members of the BIG and GBF subfamilies characterized so far function in vesicular trafficking at the Golgi [12,14,18], except for BIG2, which also localizes on recycling endosomes [19], and GNOM which acts in the endosomal recycling pathway [11].

The domain architecture of non-catalytic regions of ArfGEFs, hence their contribution to specific aspects of the build-up of the Arf response, is essentially not established except for those ArfGEFs with domains found in other classes of cellular regulators. The known domains include membrane-interacting PH domains in the CYH (reviewed in [20]), EFA6 [21] and possibly BRAG/LONER[22] subfamilies, and a putative F-box in the FBS subfamily [23], a protein-protein interaction domain that has been involved in the recruitment of substrates to the SCF ubiquitination machinery. Coiled-coil structures have also been predicted in the N-terminus of the CYH subfamily and in the C-terminus of the EFA6 subfamily. In CYH, they are involved in dimerization [3], recruitment of partners [24] and Golgi targeting [25], and in actin remodeling functions in the case of EFA6 [21]. On the other hand, although the functions of BIG and GBF subfamilies have been the subject of many investigations, their architecture is barely described, making it difficult to associate biochemical activities with their molecular structure.

Here we investigate the domain architecture in the BIG and GBF subfamilies, including all sequences from fully annotated eukaryotic genomes and our novel annotation of Sec7-containing proteins from the *Paramecium tetraurelia *alveolate. Sequence comparisons combined with secondary structures and splicing patterns analysis identifies five novel domains that are conserved between BIG and GBF subfamilies, thus unifying them as a higher order subfamily with a probable common ancestor. Our analysis of Sec7-domain containing sequences from *Paramecium *also introduces a novel subfamily of ArfGEFs unique to alveolates, which we call TBS (TBC-Sec7).

## Results and discussion

### A conserved domain architecture in BIG and GBF subfamilies

**Figure 1:**
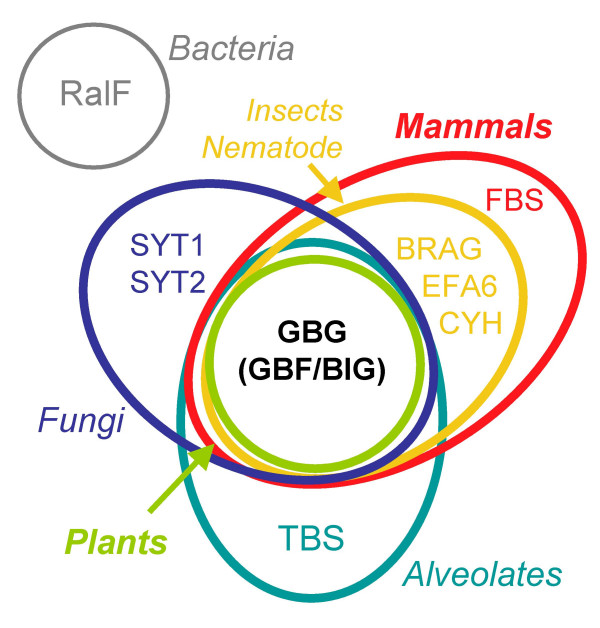
**Venn diagram of the nine Sec7-containing subfamilies sorted according to the species where each subfamily has been found. **The TBS subfamily was identified in this study. The BIG and GBF subfamilies are merged in a higher order subfamily (GBG), and are the only subfamily common to all eukaryotes.

The BIG and GBF subfamilies are the only ArfGEFs subfamilies common to all eukaryotes [8] and the sole ArfGEFs present in plants [26] (Figure 1). They are therefore possible representatives of ancestral ArfGEF functions and may provide a model to understand the nature and implementation of activities associated with the exchange function carried by the conserved Sec7 domain. However, domain 'hunting' in BIG and GBF subfamilies was complicated by the facts that the Sec7 domain is their only domain that could be identified from known domain repertoires, and that their poorly characterized non-catalytic regions were not found outside these ArfGEF subfamilies. Alternatively, we based our search of candidate structural domains in BIGs and GBFs on the bioinformatics analysis of their own sequences, taking advantage of the growing number of sequences from fully annotated genomes from mammals, insects, plants, nematode, and fungi, to which we included our annotation of Sec7-containing proteins from the newly sequenced genome of Paramecium.

**Figure 2:**
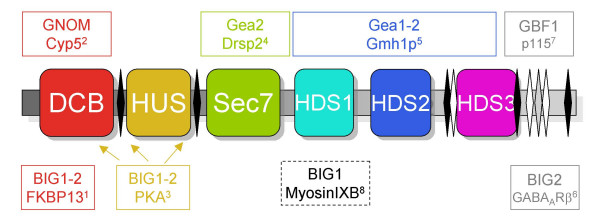
**The common domain architecture of the BIG and GBF subfamilies**. From N- to C-terminus : DCB , HUS, Sec7, HDS1, HDS2, HDS3. Linker regions of variable length and sequence are shown in grey, with alternate splicing sites in human GBF1, BIG1 and BIG2 in black, white and grey diamond shapes respectively. Interactions reported in the litterature are indicated in boxes of width corresponding to the mapped regions, except for myosin IXb interaction which was studied only with full-length BIG1. Arrows indicate predicted Protein kinase A-anchoring motifs. ^1 ^[45]; ^2 ^[27]; ^3 ^[46]; ^4 ^[47]; ^5 ^[48]; ^6 ^[49]; ^7 ^[50]; ^8 ^[51].

**Figure 3:**
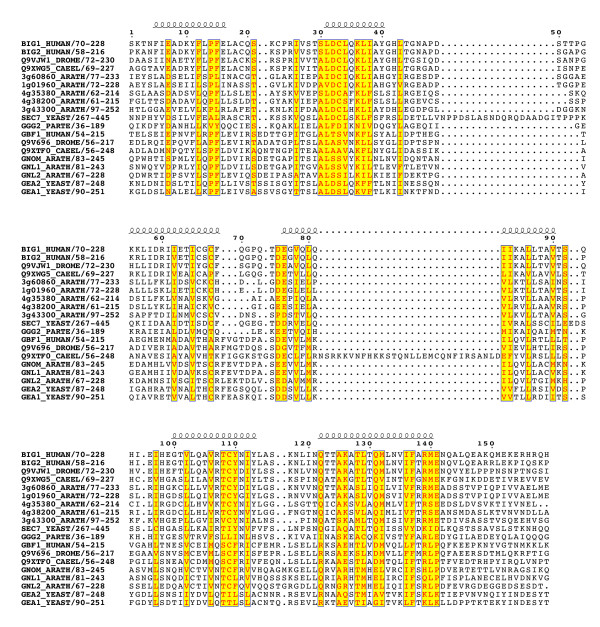
**The conserved domains of the BIG/GBF subfamily: DCB domain**. Multiple sequence alignement of the conserved domains from BIG and GBF representative sequences showing secondary structure predictions that co-align in all sequences. Colour coding is red for invariant residues, yellow for a sequence similarity score threshold of 0.15 using the BLOSUM62 matrix. The gap in helix 4 is due to an insert in the drosophila Q9V696 sequence, and may be resulting from a sequence annotation error.

**Figure 4:**
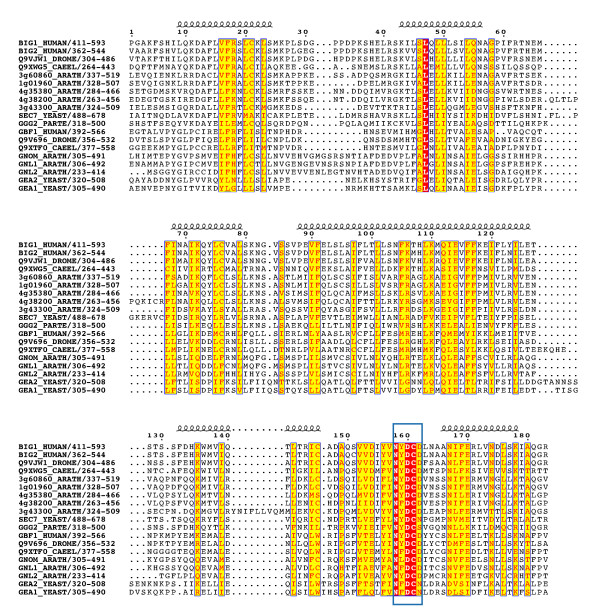
**The conserved domains of the BIG/GBF subfamily: HUS domain. **See Figure 3 legend for alignment details. The highly conserved HUS motif is boxed in blue. The gap in helix 5 domain is due to an insert in the Arabidopsis 3g43300 sequence, and may be resulting from a sequence annotation error.

**Figure 5:**
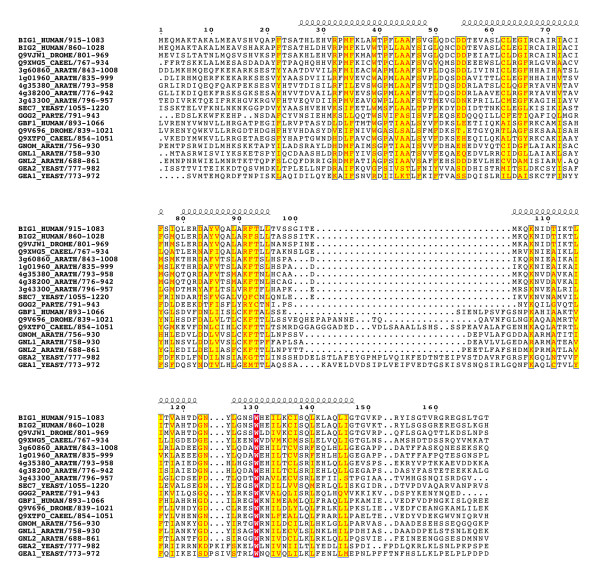
**The conserved domains of the BIG/GBF subfamily: HDS1 domain. **See Figure 3 legend for alignment details.

**Figure 6:**
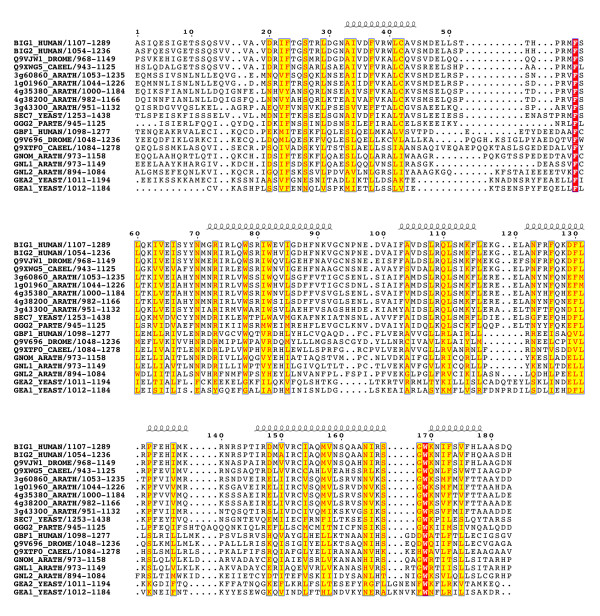
**The conserved domains of the BIG/GBF subfamily: HDS2 domain. **See Figure 3 legend for alignment details.

**Figure 7:**
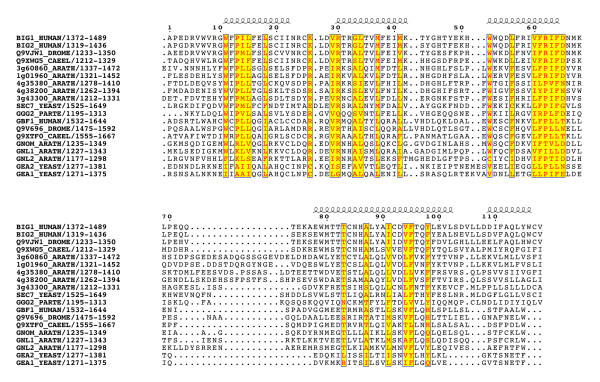
**The conserved domains of the BIG/GBF subfamily: HDS3 domain. **See Figure 3 legend for alignment details.

Multiple alignments of 42 sequences (listed in Table 1) revealed that the BIG and GBF subfamilies share an unexpected conserved architecture (schematized in Figure 2). Two homology domains are located in N-terminus of the Sec7 domain – the DCB (~150 aa) and HUS (Homology Upstream of Sec7, ~170 aa) domains – and three in its C-terminus -the HDS1 (Homology Downstream of Sec7, ~130 aa), HDS2 (~160 aa) and HDS3 (~120 aa) domains (Figure 3,4,5,6,7). In Arabidopsis GNOM, the DCB domain is included in an N-terminal region of ~250 residues involved in dimerization and possibly binding to cyclophilin5 and called the Dimerization/Cyclophilin Binding region [27], after which the new domain was named (Figure 3). All domains are predicted to have a high content of α-helices that co-align in the multiple sequence alignments, reinforcing the prediction of sequence similarities and suggesting that these domains form folded structural units that may share common functional features. Except for the N-terminal DCB domain which is also found in the yeast protein Ysl2p [28], all of them are unique to these two ArfGEFs subfamilies within the detection limits of the BLAST search. The HUS domain features a remarkably conserved N(Y/F)DC(D/N) motif, which we call the HUS box, which is predicted to locate in a loop where it may be available for functional interactions (Figure 4). The N- and C-terminal ends of BIGs and GBFs are more variable, including an unusual enrichment in Asp/Glu or Pro residues in some members. A specific feature of BIG members is that their C-terminus is in general less variable than that of GBFs, and is predicted with a significant amount of secondary structures. In contrast to the predicted structural domains, the intervening regions are highly variable in length and do not yield aligned sequences. Analysis of their amino-acid composition reveals a paucity of hydrophobic residues which is predicted to associate with an essentially unfolded conformation, suggesting that they act as linkers to tether the functional domains together.

**Table 1:** BIG and GBF protein sequences used in this study.

	Species	Protein name ^a^	Accession Number	Size in amino acids
Metazoa	Ag	Q7PWN5	EAA14874	1522
		**Q7PXQ7**	**EAA00837**	**1285**
	Ce	Q9XWG5	NP_493386	1628
		**Q9XTF0**	**NP_499522**	**1820**
	Dm	Q9VJW1	AAF53331	1653
		**Q9V696**	**AAF58532**	**1983**
	Hs	BIG1	Q9Y6D6	1849
		BIG2	Q9Y6D5	1785
		**GBF1**	**Q92538**	**1859**
	Rn	BIG1	XP_232614	1987
		BIG2	Q7TSU1	1791
		**GBF1**	**XP_347197**	**1883**

Fungi	Ca	EAL04295	EAL04295	1839
		**EAL02873**	**EAL02873**	**1015**
	Nc	Q7SAX4	EAA33549	1940
		**Q7SAL8**	**EAA33457**	**1626**
	Sc	SEC7	P11075	2009
		**GEA1**	**P47102**	**1408**
		**GEA2**	**P39993**	**1459**
	Sp	SC71	Q9UT02	1811
		SC72	Q9P7V5	1822
		**Q9P7R8**	**NP_596613**	**1462**

Viridiplantae	At	At1g01960	Q9LPC5	1750
		At3g43300	NP_189916	1728
		At3g60860	Q9LZX8	1793
		At4g35380	O65490	1711
		At4g38200	NP_195533	1698
		**GNOM**	**Q42510, At1g13980**	**1451**
		**GNL1**	**Q9FLY5, At5g39500**	**1443**
		**GNL2**	**NP_197462, At5g19160**	**1375**
	Os	9631.m01366	Q8S565	1789
		9630.m00920	Q9XGN9	1687
		9634.m04029	-	1704
		9635.m03752	-	1680
		**9631.m04495**	**-**	**1456**
		**9630.m02122**	**-**	**1396**
		**9632.m00175**	**Q7XT11**	**1407**

Alveolata	Pt	GGG1	CR533425	1615
		GGG2	CR533424	1628
		GGG3	CR533423	1598
		GGG4	CR533422	1599
		GGG5	CR533421	1435

^a ^Unnamed sequences are designated by their NCBI accession number, AGI (Arabidopsis Genome Initiative) locus numbers for At and TIGR model temporary IDs for Os. BIG and GBF subfamily members are in normal and bold characters respectively, except for Pt members which have not been assigned to either subfamily (see also Figure 8). Species abbreviations are: Ag, *Anopheles gambiae*; Ce, *Caenorhabditis. elegans*; Dm, *Drosophila melanogaster*; Hs, *Homo sapiens*; Rn, *Rattus norvegicus*; Ca, *Candida albicans*; Nc, *Neurospora crassa*; Sc, *Saccharomyces cerevisiae*; Sp, *Schizosaccharomyces pombe*; At, *Arabidopsis thaliana*; Os, *Oryza sativa*; Pt, *Parameciumtetraurelia*).

To further investigate the predicted organization of BIGs and GBFs in 6 conserved helical domains connected by variable linkers, splicing patterns of human BIGs and GBFs were analyzed in the large number of cDNAs and ESTs in the databases that correspond to GBF/BIG transcripts. This revealed the use of alternate splice donor and acceptor sites predicted to yield proteins with insertions and deletions ranging from 1 to 38 residues, and a number of splice variants arising from exon skipping (Table 2). Strikingly, all observed sequence variations occur in regions identified as linkers between conserved domains (Figure 2). Together with our domain analysis, this suggests that splicing at non-canonical exon/intron boundaries is only tolerated in regions of the protein where the impact upon folding of domains with essential function would be minimal.

**Table 2:** Alternate splice variants of human GBF1, BIG1 and BIG2 ^a,b^

	**Change in protein**	**Apparent cause of variation in transcript**
		
GBF1	Extra Q at 337, 55 residues upstream of HUS domain	Insertion of 3 nucleotides (nt) resulting from use of alternate 3' acceptor site within intron during splicing of exons 10 and 11
	New Ser and loss of 14 residues at 613, between HUS and Sec7 domains	Loss of 36 nt resulting from use of alternate 5' donor site within exon 15 during splicing with exon 16
	Loss of VSQD at 1494, 38 residues upstream of HDS3	Loss of 12 nt resulting from use of alternate 5' donor site within exon 33 during splicing with exon 34
	Frame-shift at 1625 causing loss of last 19 residues of HDS3	Intron retention between exons 36 and 37 leading to frame shift and premature termination
	Loss of 38 residues starting at 1784, near C-terminus	Loss of 114 nt resulting from use of novel cryptic splice donor and acceptor sites within exon 40.
		
BIG1	Frame-shift at 1340, 32 residues upstream of HDS3	Loss of 59 nt resulting from use of alternate 5' donor site within exon 28 during splicing with exon 29
	Loss of VSEKPL at 1557, 68 residues downstream of HDS3	Loss of 18 nt resulting from use of alternate 5' donor site within exon 33 during splicing to exon 34
	New T and loss of 33 residues at 1607, 118 residues downstream of HDS3	Loss of 96 nt resulting from use of alternate 3' acceptor site within exon 35 during splicing with exon 34
		
BIG2	Frame-shift at 1542, 106 residues downstream of HDS3	Loss of exon 35 resulting from splicing of 5'donor site of exon 34 with 3' acceptor site of exon 36

^**a **^All changes were expressed relative to the reference sequence stored under accession number NM_004193 (hGBF1), NM_006421 (hBIG1) and NM_006420.1 (hBIG2).

^**b **^All variants are supported by one or more cDNA/ESTs as detailed in the Aceview for each gene that can be obtained at [38].

### Evolution of BIGs and GFBs from a common ancestor

**Figure 8:**
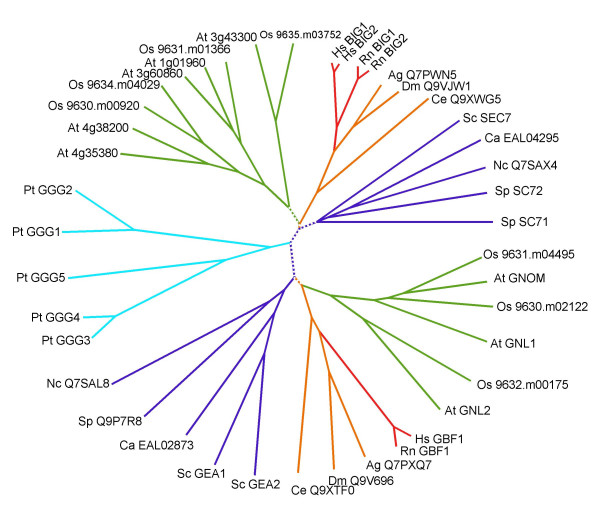
**Unrooted neighbour-joining phylogenetic tree of the BIG/GBF subfamily. **Colour coding for the main groups is green for plants, marine blue for fungi, orange and red for animals, cyan for protists. Branches found in less than 60% bootstrap trials by either the neighbor-joining or the maximum likelihood method are in dotted lines. Species abbreviations are as in Table 1.

Combined, our analysis reveals that the BIG and GBF subfamilies share the same overall domain organization, and are likely to descend from a common ancestor gene that duplicated first to form the BIG and GBF groups, and again within these groups to yield species-specific BIG and GBF members. These two subfamilies can therefore be unified as a higher order ArfGEF subfamily (called below GBG for GBF/BIG GEFs), from which unrooted phylogenetic trees can be built (Figure 8). Unlike previous phylogenetic analysis which compared ArfGEFs based on their Sec7 domains after diverging non-catalytic regions have been trimmed [8], our trees were established from the simultaneous alignment of all 6 conserved domains (DCB, HUS, Sec7, HDS1, HDS2 and HDS3), excluding variable linkers. The same tree topology was obtained with both neighbor-joining and maximum likelihood methods, and was retained using any one of the new conserved domains alone (data not shown). Bootstrap analysis strongly supported this topology for most branches. Only a few small branches located at the base of the groups were found in less than 60% of the trials in one of the two methods, but this never occurred with both methods simultaneously.

The tree topology strongly suggests that in most organisms, GBG members sort in separate branches, corresponding to their classification in BIGs and GBFs. Remarkably, our annotation of Sec7-containing proteins in the genome of Paramecium reveals the first departure from this distribution, as all GBGs in this species are located in a single branch, which is closer to the BIGs. This unexpected tree topology may indicate that alveolates diverged from animals/fungi and plants before the duplication of an ancestral GBG into the BIG and GBF, and that GBGs in that organism are representative of this ancestral gene. Alternatively, duplication may have been followed by loss of GBF genes. Current knowledge of the phylogenetic branching of alveolates relative to the plants and animal/fungi branches does not permit resolution between those two possibilities.

### GBGs in plants: refining the functional evolution of BIGs and GBFs

In fungi and mammals, BIGs and GBFs are represented by only one or two members, whose functions in vesicular trafficking at the Golgi within each group appear largely overlapping [12,14]. In contrast, plants encode a large number of GBGs in both the BIG and GBF branches but lack other ArfGEFs (Figures 1, 8). In Arabidopsis, none of the GBGs map to duplicated chromosomes where identical functions may be encoded [26,29]. In addition, comparative analysis with the rice genome nearing completion identifies at least five branches each represented by one rice and one or two Arabidopsis homologs (Figure 8). This correspondence between two highly divergent plant species indicates that GBGs diversified early during plant evolution, probably reflecting functional specialization along with the establishment of plant multicellularity. While GNOM has a plant-specific function in recycling plasma-membrane proteins needed for cell-cell communication and cell polarity establishment [11], possibly closer to the function of EFA6 or CYH subfamilies in metazoans, other plant GBGs are expected to fulfill the presumed ancient function of regulating Golgi trafficking exemplified by mammalian and yeast GBGs. Comparison of orthologous pairs in plants further reveals that they have different sensitivities to Brefeldin A (a widely used fungal inhibitor of Golgi traffic) as predicted from the sequences of the binding site of the drug carried by the Sec7 domain [6]. This observation clearly illustrates that differences in outcome following BrefeldinA treatment may not reflect differences in underlying molecular mechanisms, but instead simply reflect neutral sequence differences at the Sec7 domains between species. In particular, not all BIGs may be BFA-sensitive or GBFs BFA-resistant, unlike suggested by their original nomenclature.

### A novel ArfGEF subfamily in alveolates

**Figure 9:**
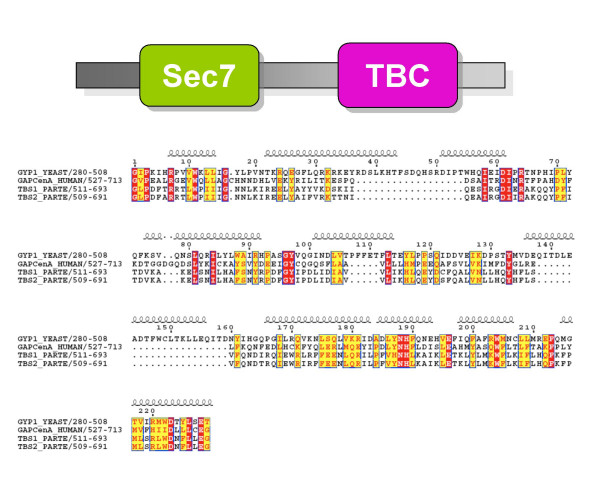
**TBS: a novel ArfGEF subfamily in alveolates. **Top: Domain structure of the TBS subfamily. Below: Sequences of the TBC domain from Paramecium TBS aligned with TBC domains from known RabGAPs. Secondary structures are from the crystal structure of yeast GYP1 [30].

A remarkable evolutionary feature of ArfGEFs is that while GBGs seem to be ubiquitous to all eukaryotes, fungi and animals kingdoms evolved their own ArfGEFs subfamilies unrelated to those of the other kingdoms. We thus addressed the question of whether Paramecium, which has a large number of GBGs (at least five, of which four are present as pairs as the result of recent duplications) but appears to lack the specialization into the BIG and GBF subgroups, has the same ArfGEF distribution as plants or features a second ArfGEF subfamily. We thus searched the newly sequenced genome from *Paramecium tetraurelia *and the available alveolate genomes from *Cryptosporidium parvum *and *Tetrahymena thermophila *for additional Sec7-containing proteins. This identified a novel putative ArfGEF subfamily characterized by the association of the Sec7 domain with a TBC (Tre/Bub2/Cdc16) domain (Figure 9), which was found only in the protists kingdom. The TBC domain is predicted to carry a GAP (GTPase activating protein) activity towards small G proteins of the Rab family [30], suggesting a potential crosstalk between Rab and Arf pathways. Such a relationship between these two small G proteins families, which are major regulators of membrane traffic, would not be unprecedented, as for example the SYT1 ArfGEF gene was identified in yeast by its genetic interactions with Rab proteins in the exocytic pathway [31]. Interestingly, alveolates have specialized exocytic pathways based on a membrane organelle lying beneath the plasma membrane, the trichocyst, where this unique ArfGEF family may potentially function.

## Conclusion

### A conserved scenario for the activation of Arf proteins by their GEFs?

The identification of a conserved modular architecture in all GBG subfamily members suggests that the mechanistic basis for their activation of Arf is likely to follow a similar scenario. Candidate functions for the conserved domains include oligomerization, the collection of input signals, membrane localization, regulation of the exchange activity, scaffolding of Arf proteins to their downstream effectors, not excluding signaling to partners outside the Arf pathways. Dimerization has been reported in the BIG subgroup for BIG1, which forms heterodimers with the highly homologous BIG2 ArfGEF [14], and in the GBF subgroup for GNOM, which forms homodimers [27]. The conservation of the DCB domain in GBGs, which is responsible for the dimerization of GNOM, suggests that such a dimerization function may be general to this domain in GBGs. Another unresolved issue is the conservation of the cellular partners effecting the functions associated with the conserved domains. Our identification of an almost invariant motif in the HUS domain argues in favor of this domain interacting with a conserved partner. However, the ancient divergence into the BIG and GBF groups and their subsequent divergence into species-specific members suggest that specialized requirements are likely to have evolved in most organisms, possibly yielding less conserved partners outside the Sec7 and HUS domains. Finally, whereas in plants all ArfGEFs are predicted to function according to the scheme defined by the conserved domains, other species have additional ArfGEF subfamilies with a modular architecture unrelated to that of the GBG subfamily. It is not known to what extent the GBG's scenario for Arf activation will also apply to non-GBGs ArfGEFs, acting alone or in association with protein partners. In the case of the GBGs, our definition of the structural homology domains as reported here should now provide a robust background for future investigations of their interactions and functions.

## Methods

Protein sequence databases were searched with amino acid sequences from human BIG1, human GBF1 and Arabidopsis GNOM using the BLAST algorithm [32]. *Paramecium tetraurelia *genes were identified with the BLAST algorithm using genome sequence data from Genoscope [33] and manually annotated using Artemis [34]. Tetrahymena sequences were retrieved from the *Tetrahymena thermophila *genome sequencing project server [35]. Arabidopsis sequences were retrieved from the Arabidopsis Genome Initiative database [36], rice sequences from the TIGR Rice annotation project [37]. Splice variants for hGBF1, hBIG1 and hBIG2 were identified from information provided under Aceview in the December (03) release for their respective listings at the NCBI [38]. Multiple sequence alignments were performed using ClustalW [39] with default alignment parameters or T-coffee [40,41]. Reliability of the alignments was evaluated according to the T-coffee score, and ranged from average to good for all predicted domains. Average sequence identities were respectively 24 % (DCB domain), 26 % (HUS domain), 44% (Sec7 domain), 26% (HDS1 domain), 28% (HDS2 domain) and 21% (HDS3 domain). Aligned sequences were displayed with ESPript [42] using a similarity global score of 0.15 calculated using the BLOSUM62 matrix. Unrooted phylogenetic trees were generated using the neighbor-joining algorithm of ClustalW excluding gapped regions, and with a maximum likelihood method using the PHYML package [43]. Phylogenetic trees for individual domains was performed on the subset of sequences used in Figure 3. The reliability of the trees was assessed by a bootstrap analysis (1000 replicates). Trees were drawn with TreeView version 1.6.6. Secondary structure predictions on aligned sequences were carried out with the PHD program along with the ClustalW multiple alignment [39]. Non-structured linkers poor in hydrophobic residues were predicted with the PONDR algorithm [44].

## Abbreviations

GEF: Guanine nucleotide exchange factor. CYH: cytohesins/ARNO; EFA: Exchange Factor for Arf6; FBS: F-Box/Sec7; TBS: TBC/Sec7; GBF: Golgi-associated BFA-resistant guanine nucleotide exchange Factor; BIG: BFA-Inhibited Guanine nucleotide exchange factor; GBG: GBF/BIG Gefs; SYT1: *S*uppressor of *ypt*. DCB: Dimerization/Cyclophilin Binding; HUS: Homology Upstream of Sec7; HDS: Homology Downstream of Sec7; TBC: Tre/Bub2/Cdc16; SCF: Skp1/Cull1/F box.

## Authors' contributions

B.M. and V.B. carried out sequence and phylogenetic analysis. A.J. participated in the domain analysis. J.Co. annotated Paramecium sequences. D.S. and P.M. performed splicing pattern analysis. N.K. and G.J. analyzed the distribution of large ArfGEFs in plants. J.Ch. conceived and coordinated the study and wrote the manuscript.
